# Lower between-limb asymmetry during running on treadmill compared to overground in subjects with laterally pronounced knee osteoarthritis

**DOI:** 10.1371/journal.pone.0205191

**Published:** 2018-10-18

**Authors:** Jacques Robadey, Didier Staudenmann, Raphael Schween, Dominic Gehring, Albert Gollhofer, Wolfgang Taube

**Affiliations:** 1 Movement and Sport Science, Department of Medicine, University of Fribourg, Fribourg, Switzerland; 2 ICT Department, University of Applied Sciences and Arts, Western Switzerland, Fribourg, Switzerland; 3 Department of Sport Sciences, University of Freiburg, Freiburg, Germany; 4 Institute of Sport Sciences, University of Giessen, Giessen, Germany; Sao Paulo State University - UNESP, BRAZIL

## Abstract

Subjects with knee osteoarthritis (KOA) show gait asymmetries evidenced by lower knee flexion and shorter contact times for the affected leg. Interestingly, running on a treadmill compared to running overground is also associated with lower knee flexion and shorter contact times. Thus, it is of particular interest how gait patterns are influenced by the type of ground in subjects with KOA. The aim of the current study was therefore to measure the overground asymmetry of kinematic parameters in KOA subjects while running and to investigate whether this asymmetry is altered on a treadmill. Nine patients diagnosed with KOA underwent overground and treadmill running with 3D-motion analysis. The symmetry analysis was performed using Symmetry Angles for five selected gait parameters: contact and step time, heel-toe delay, maximal knee flexion during stance and vertical speed variance. For all parameters, the values were significantly lower for the affected compared to the non-affected leg (p≤0.023). Post-hoc analyses revealed significant differences between legs only overground and not on the treadmill. The asymmetry was lower on the treadmill, as indicated by significant Symmetry Angle reductions for contact time (p = 0.033), knee flexion (p = 0.001) and vertical speed variance (p = 0.002). The symmetry increase on the treadmill was mainly due to changes of the non-affected leg towards the affected leg values leading to smaller steps and less impact load in general. The present results suggest therefore that a) an assessment of symmetry may differ depending on the ground type (treadmill versus overground) and b) treadmill running may be more suitable for patients with KOA related gait asymmetries.

## 1. Introduction

There is good evidence about altered gait kinematics in subjects suffering from chronic knee osteoarthritis (KOA) when tested during walking. For instance, greater stride duration [1–3] and lower gait velocity [3, 4], knee flexion [2, 3, 5], peak knee flexion moments, peak hip adduction moments and peak hip extension moments were found during walking [6]. As these changes are more pronounced in the injured leg, they have been shown to induce inter-limb asymmetries for KOA subjects during walking [7].

For running, however, there is, to our knowledge, currently no evidence about the influence of KOA on between-limb symmetry. So far, it has been demonstrated that participants with a non KOA knee injury display lower mid-stance knee flexion [8, 9], larger pelvis tilt and more hip internal rotation than healthy controls while running [10]. Leg-injured runners also showed larger asymmetries in contact time than healthy controls [11]. Based on these examples, we speculate that subjects with a chronic KOA also display altered gait parameters resulting in asymmetries during running. To verify this assumption, the first goal of this study was to assess running patterns of physically active participants with KOA. During running, we expected lower knee flexion and shorter contact times for the affected leg as already observed for walking [12].

Interestingly, running on a treadmill (TM) compared to running overground (OG) is also associated with lower knee flexion [13] as well as shorter contact and step times [14, 15]. Thus, it is of particular interest how gait patterns are influenced by ground types in subjects with KOA. So far, no study compared the running gait asymmetry between OG and TM locomotion. This might be problematic, as to our knowledge, all previous studies about leg injury patients have investigated gait and running patterns either exclusively on TM [1, 9, 11] or exclusively OG [2, 7, 10] without ensuring the comparability of the assessment of asymmetry between both ground cases. This may surprise, as there is accumulating evidence that locomotion on the TM and OG differs. For instance, several studies in stroke patients have demonstrated increased symmetry during walking on the TM compared to walking OG [16, 17]. In addition, running on a TM displayed more regular stride timing dynamics than running OG [18]. Furthermore, based on differences in muscular activity between OG and TM, Oliveira et al. [19] assumed higher demands for load absorption at initial contact for OG compared to TM-running. Moreover, a flatter foot-landing position lowering the heel-toe delay [20, 21], a reduction in step length [22], step time, contact time [23], vertical speed variance [14, 23] and maximal knee flexion during the stance phase [13] were found when subjects switched from OG to TM. As these adaptations on the TM are independent from gait-alterations due to knee injury, but nevertheless represent some similarities with adaptations observed OG for the injured leg of KOA patients, the second and main goal of the current study was to clarify whether TM locomotion alters the asymmetry-pattern of patients with KOA.

For this purpose, we assessed OG and TM running characteristics of KOA patients with a special focus on gait symmetry. Kinematic gait parameters were analysed for steps performed with the affected leg (AL) and the non-affected leg (NL) on TM and OG. The symmetry of these parameters was expressed by using the so-called Symmetry Angle (SA) [24]. Based on the literature mentioned above [2, 6, 13], we hypothesized a reduction of the running-asymmetry on the TM compared to OG.

## 2. Methods

Data for this study were obtained in the same experiment as those published previously in Schween et al. [25]. We here describe the methods to the extent they are relevant for the current analysis. The experiment was conducted in accordance with the Declaration of Helsinki and approved by the ethics committee of the University of Freiburg, Germany (107/12). Before testing, all participants gave written, informed consent.

### 2.1 Subjects and procedure

Inclusion criterion to the experiment was a medically diagnosed KOA. All the investigated subjects had a unilateral injury. The KOA injured leg was called “affected leg" AL and the other one “non-affected leg” (NL). Subjects’s Kellgren-Lawrence grades were between 2 and 3 (mean 2.8 ±0.4). Subjects with neurological disorders, prosthetic implants and any condition contraindicating the physiological demands of the experiment were excluded. Out of 19 participants that practiced a sport activity at least twice per week, nine were selected for this analysis, based on the criteria being clear heel runners (6 women and 3 men; mean ± standard deviation: 50±9 years; mass: 64.6 ± 9.6 kg; body size: 170.1 ± 9.5 cm). Seven subjects were excluded as they did not show a running pattern (some steps showed no flight phase), while three subjects were not considered as they displayed a forefoot running strategy.

Prior to the experiment, the subjects walked for about 5 minutes before running OG for 1 minute at their natural speed. During the last part of this warm-up process, the natural running speed was determined with light barriers for each subject. In the actual experiment, subjects were tested at this self-selected running speed for both OG and TM conditions. The order of ground types (TM or OG) was randomized to level out fatiguing effects or any other systematic bias. At least ten trials of 10 m corresponding to about ten steps (five on both legs) were captured OG at target speed ±0.05 m/s. This was done by immediately assessing speed with light barriers and repeating trials outside these limits. In this case, participants were informed in which direction they had to adjust their speed. The ground forces were measured for both legs on separate force plates for each trial. On the TM, subjects ran a single bout of about 3 minutes with the TM speed set to their mean speed as determined during warm-up. After one minute familiarization, 5 trials of 20 seconds (about 50 steps) were captured.

With the above-described procedure, about 100 OG and 200 TM steps were captured and analysed for each subject. Each step, and by that way both legs, were analysed in each trial. This procedure reduces any effects that fatigue might have had on the between-leg comparison, since fatigue would be expected to affect both legs. Furthermore, the current analysis consists of the measurement of specific parameters (presented in the “data analysis” section) on the base of all trials. The final parameters correspond to the arithmetic average of the measurements from all trials and that for OG and TM, and for AL and NL.

Standardized shoes (Spezial, Adidas, Herzogenaurach, Germany) with low cushioning and no custom insoles were worn throughout the experiment to prevent potential footwear-related effects.

### 2.2 Equipment and data collection

The kinematics of the lower extremity were assessed using a 3D-motion analysis system (Vicon V-MX, VICON Motion Systems Ltd., Oxford, UK) at a sampling frequency of 200Hz by placing reflecting markers on the pelvis, thigh, lateral and medial epicondyle of the knee, both malleoli, shanks and feet (in line with [25]). The VICON system has a marker position precision of about 2mm [26] and a time precision of about 2.5 ms due to its sampling rate. Overground, we measured the average running speed using light barriers (Timer S3, ALGE, Maienfeld, Switzerland; with a precision below 0.001 m/s) and the ground reaction force with a force plate (BP600900-2000, Advanced Mechanical Technology Inc., Watertown, MA, USA) at a sample rate of 2000Hz. The used treadmill (quasar 5.0, h/p/cosmos sports and medical GmbH, Nussdorf-Traunstein, Germany) was new and was, prior to the experiment, directly installed by the equipment provider. No specific speed and slope calibration [27] was therefore required. The slope was set at 0% by a motorized adjustment and the naturally selected speed was programmed with a precision of 0.1m/s. Kinematic data was synchronized to ground reaction forces in the OG condition and both signals were low-pass filtered at 15Hz (4^th^ order Butterworth, bidirectional, in line with Schween et al. [25].

### 2.3 Data analysis

We selected those kinematic parameters for our analysis that had previously been shown to differ between KOA-patients and healthy controls [1–5] as well as between OG and TM locomotion [13, 14]. These were *a) contact time*, *b) step time*, *c) heel-toe-delay*, and *e) vertical speed variance*. Data were analysed with Matlab (The Mathworks, Inc., Natick, Massachusetts, USA) to determine the kinematic parameters with high reliability as follows [28]: The arithmetic average of the four pelvis markers placed at the left respective right posterior and anterior superior iliac spine was used to define the centre of pelvis (CP), which can be considered as an approximation of the centre of mass [29]. CP velocity and acceleration were obtained by derivation of the CP position in the sagittal plane.

**Fig 1:**
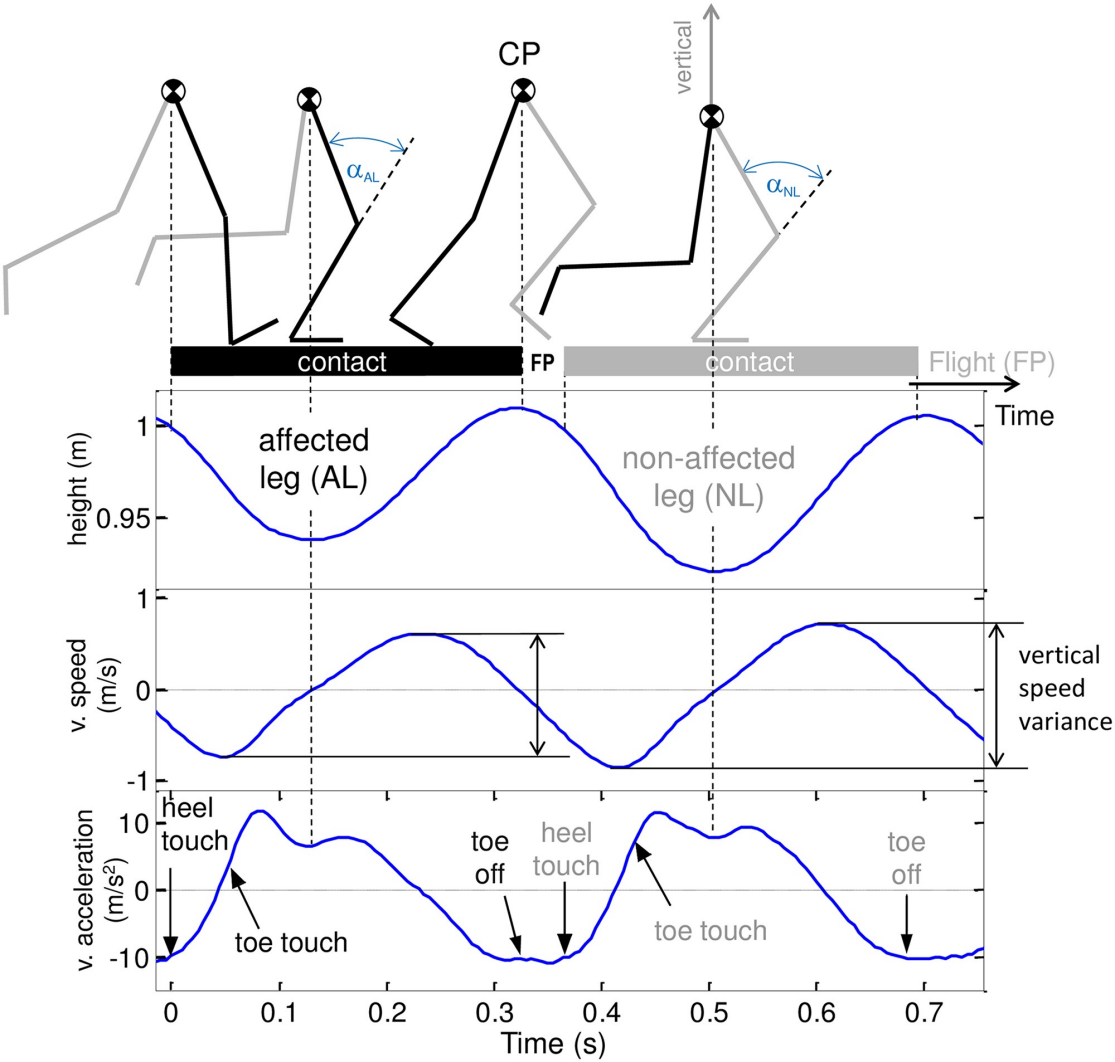
Running phases with maximal knee flexion and centre of pelvis vertical movement as a function of time. The top panel is a schematic representation of a human running in sagittal plane with contact and flight phases. CP represents the centre of pelvis and α the maximal knee flexion during stance for the affected and the non-affected leg (AL, NL). The lower three panels show the CP height, vertical speed and vertical acceleration measured during an overground (OG) gait cycle for one KOA patient. The vertical speed variance corresponds to the difference between minimal and maximal vertical speed during stance (illustrated for both AL and NL). Note the larger vertical movement and speed variance for NL.

1. The *contact time (t*_*c*_*)* was defined as the time between touch-down and take-off, which were assessed OG by force plate signals. For the TM case, we first measured OG the individual average delay between touch-down and time of minimal heel elevation for each subject and each leg. This delay was due to the small skin and marker displacement relative to the calcaneus. Second, we obtained the time of touch-down on the TM by subtracting this delay from the minimal heel elevation time. The TM take-off time was determined in a similar way: the average delay between the time of lowest toe elevation and take-off (determined by the abrupt nullification of the force plate signal) was measured OG. This delay was added to the TM time of lowest toe elevation in order to obtain the TM take-off time.
2. The *step time (t*_*s*_*)* of the AL was defined as the time between the touch-down of AL and the touch-down of the NL and vice versa.
3. Furthermore the *heel-toe delay (HTD)* was defined as the time between touch-down and toe touch (corresponding to an abrupt cessation of the toe marker vertical velocity).
4. The *maximal knee flexion (α)* during ground contact was determined by analysing the respective joint angle for each measured time frame and selecting the minimal knees angle obtained during the stance phase.
5. Finally, we determined the *vertical speed variance (VSV)* as the peak-to-peak variation of the CP vertical velocity during the stance phase (Fig 1). All mentioned parameters were assessed for both legs (NL/AL) and for both OG and TM cases. Subsequently, the between-leg differences of each parameter: Δ (X_NL_–X_AL_) were calculated for OG and TM.

For an assessment of the bilateral symmetry of each kinematic parameter, we used the Symmetry Angle from Zifchock et al., [11, 24]:

$$SA=\frac{45{}^{\circ}-\text{arctan}\left(X_{AL}/X_{NL}\right)}{90{}^{\circ}}∙100$$ (1)

with *X*_*AL*_/*X*_*NL*_ being the parameter ratio for AL and NL. Note that in Eq 1, an SA value of zero indicates perfect symmetry between the NL and AL. In contrast to the symmetry index [30], the SA is not prone to problems of normalization and does not require an adequate reference value [24].

### 2.4 Statistics

The parameters: t_c_, t_s_, HTD, α and VSV were statistically tested with separate two-way repeated measure analysis of variance (SPSS 19, Inc. Chicago, IL, USA), taking into account the LEG (NL/AL) and GROUND (OG/TM) factors. Subsequently, post-hoc tests were applied by using Bonferroni-corrected paired Student’s *t*-tests to specify differences between NL and AL for each ground type and between OG and TM for each leg.

Symmetry Angles for each independent parameter were compared between OG and TM using paired Student’s *t*-tests. The two-sided significance level was set at 5% and data are reported as means ± standard deviations in the text.

## 3. Results

**Fig 2:**
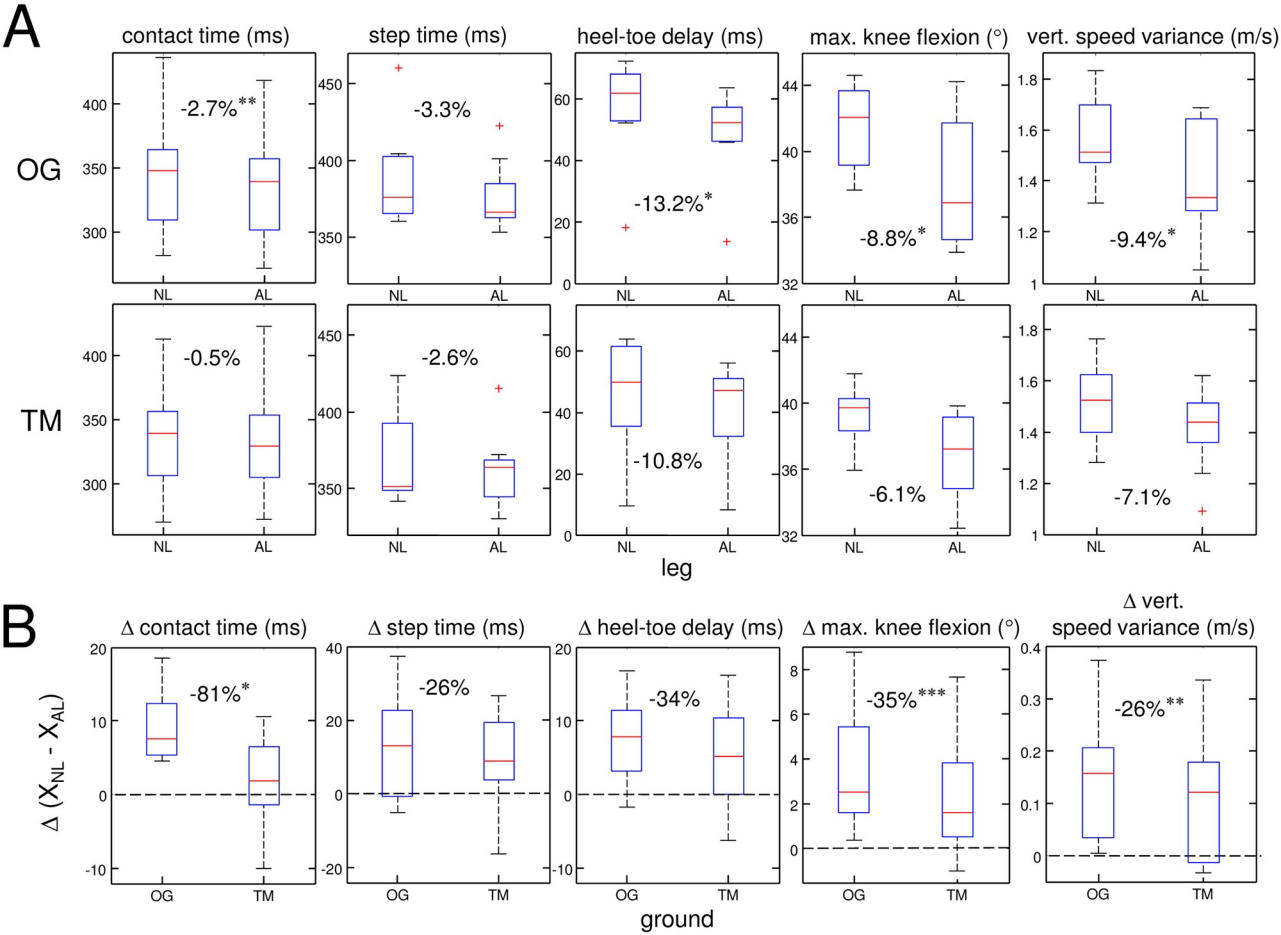
Kinematic parameters side dependency for OG and TM ground. A) Overall results of the kinematic parameters for overgound (OG) and treadmill (TM) running. NL refers to the non-affected leg and AL to the affected leg. Boxplots show the results over all subjects. For each boxplot, the middle line represents the median value, the lower and upper limits represent the interquartile range and the error bars indicate the range and the plus signs denote outliers. Stars (*, **, ***) indicate significant differences (p<0.05, p<0.01, p<0.001) between NL and AL. Note that OG the differences between the NL and the AL were larger for all kinematic parameters compared to when running on the TM. B) Difference between the NL and AL value for all parameters. With dotted lines representing full symmetry, a symmetry increase is observed for all parameters.

The average running velocity, took identical values of 2.4 ± 0.3 m/s for both OG and TM. Results of the five kinematic parameters with their NL/AL side dependency are shown in Fig 2 for both ground types. In paragraph 3.1, the differences between NL and AL, and between OG and TM are presented together with the interaction between LEG and GROUND factors. The Symmetry Angles of each kinematic parameter are described in paragraph 3.2.

### 3.1 Kinematic parameters

We found significant main effects for LEG, with AL displaying lower values than NL for all parameters as illustrated in Fig 2: t_c_ (-1.6%, *F*_1,8_ = 45.4, *p* < 0.0002), t_s_ (-2.9%, *F*_1,8_ = 7.84, *p* = 0.023), HTD (-12.2%, *F*_1,8_ = 9.16, *p* = 0.016), α (-7.5%, *F*_1,8_ = 10.9, *p* = 0.011) and VSV (-8.3%, *F*_1,8_ = 9.95, *p* = 0.013). On the other hand, the GROUND condition showed a significant reduction on TM only for the step time and the maximal knee flexion: t_s_ (-4.2%, *F*_1,8_ = 49.3, *p* < 0.0001) and α (-4.5%, *F*_1,8_ = 6.8, *p* = 0.03) while a trend towards lower TM values was observed for HTD (-19.3%, *F*_1,8_ = 4.72, *p* = 0.061).

The contact time, maximal knee flexion and vertical speed variance revealed significant interactions between LEG and GROUND factors: t_c_ (*F*_1,8_ = 5.67, *p* = 0.044), α (*F*_1,8_ = 25.4; *p* < 0.001) and VSV (*F*_1,8_ = 19.7, *p* = 0.002).

Interestingly, the post-hoc analyses of all parameters (except the step time) revealed significant reduction for the AL only OG and not on TM (Fig 2): t_c_ (OG—2.7%, *t*_8_ = 5.94, *p* = 0.0014; TM:—0.5%, *t*_8_ = 0.88, *p* > 0.9), HTD (OG: -13.2%, *t*_8_ = 3.83, *p* = 0.02; TM: -10.8%, *t*_8_ = 2.04, *p* = 0.3), α (OG: -8.8%, *t*_8_ = 3.91, *p* = 0.018; TM: -6.1%, *t*_8_ = 2.66, *p* = 0.12) and VSV (OG: -9.4%, *t*_8_ = 5.59, *p* = 0.028; TM: -7.1%, *t*_8_ = 2.68, *p* = 0.11).

#### Between leg differences of kinematic parameters

All parameters showed a reduction of their between leg difference: Δ = (X_NL_–X_AL_) when switching from OG to TM (see Fig 2b) with significant reductions observed for t_c_, α and VSV. The between leg difference of t_c_ almost disappeared on the TM while for the other parameters, Δ was reduced by roughly 30%.

### 3.2 Symmetry analysis

**Fig 3:**
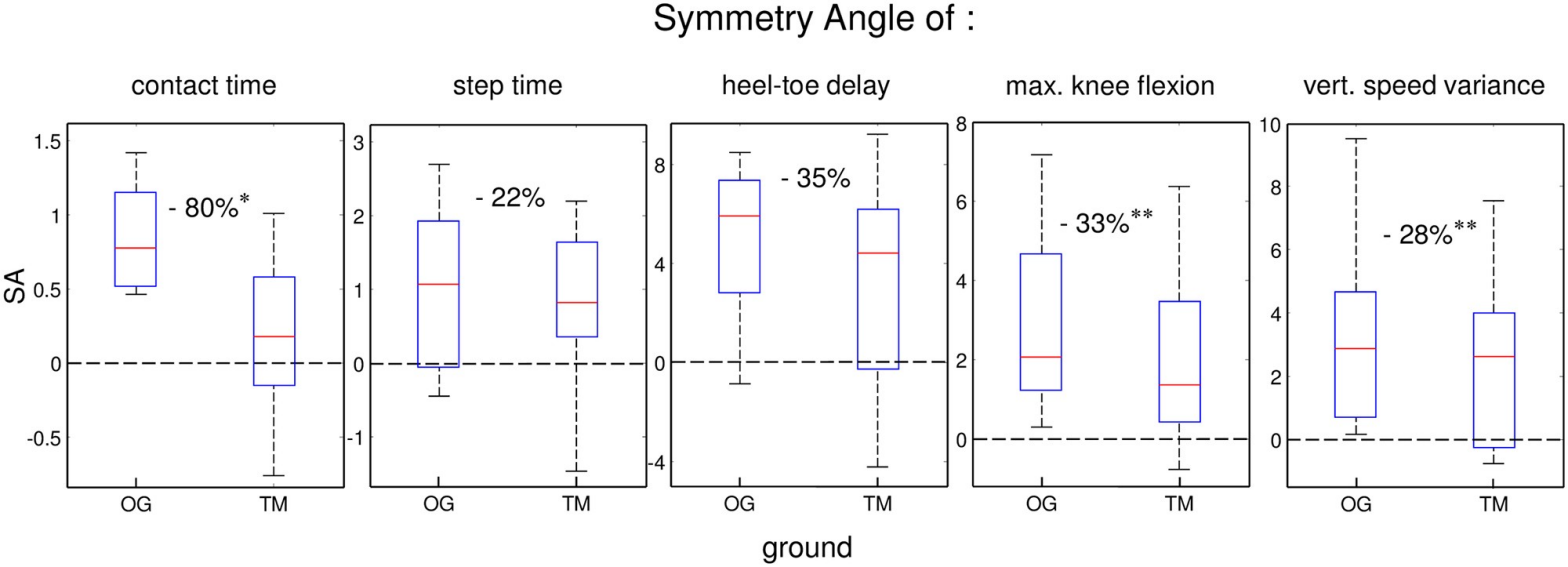
Overall results of the Symmetry Angle for overground (OG) and treadmill (TM). Boxplots show the results over all subjects with dotted lines corresponding to lines of full symmetry and stars indicating significant differences. We observe a Symmetry Angle decrease on TM compared to OG for all parameters, revealing a general symmetry increase on the TM.

We calculated the Symmetry Angle for all kinematic parameters using Eq (1) for both OG and TM, and show the results in Fig 3. Symmetry was significantly increased when subjects switched from OG to TM indicated by a reduced SA for contact time (-80%; OG: 0.86 ± 0.38; TM: 0.17± 0.52; *t*_8_ = 2.58, *p* = 0.033), maximal knee flexion (-33%; OG: 3.0 ± 2.4; TM: 2.0 ± 2.3; *t*_8_ = 4.96, *p* = 0.001) and vertical speed variance (-28%; OG: 3.3 ± 3.1; TM: 2.4 ± 2.8; *t*_8_ = 4.38, *p* = 0.002). The Symmetry Angles for step time (OG: 1.0 ± 1.2; TM: 0.8 ± 1.1; *t*_8_ = 0.52, *p* = 0.62) and heel-toe delay (OG: 4.8 ± 3.3; TM: 3.1 ± 4.6; *t*_8_ = 0.97, *p* = 0.36) displayed no differences between ground conditions. These results thus corroborate those based on between-leg differences.

## 4. Discussion

The present study demonstrated significant differences between the ALs and NLs when measured OG. However, these asymmetries were effectively reduced when running was performed on TM.

### Differences between affected and non-affected leg overground

Previous studies compared the affected legs of runners with various knee injuries to the unaffected legs of healthy controls [8, 9, 31, 32]. In that respect, knee-injured subjects displayed lower knee flexion than non-injured subjects [8, 9]. These differences between injured and healthy subjects are in line with the differences between the AL and the NL observed OG in the current study: a significantly smaller knee flexion occurred in the AL than the NL. Furthermore, all other parameters except step time, thus, contact time, heel-toe delay and vertical speed variance, also displayed significant differences between AL and NL when measured OG (see Fig 2). Interestingly, large differences between the AL and the NL of about 10% were found OG for maximal knee flexion, vertical speed variance and heel-toe delay. All these parameters were associated with high (near maximal) vertical movement dynamics, i.e., ground reaction forces. Indeed we find in Fig 1 as previously observed [33] a nearly maximal ground reaction force (F = m*a, assuming that the lower panel Pelvis acceleration “a” is a good approximation of the centre of mass acceleration) at the time of maximal knee flexion (dotted lines). In contrast, step time and contact time, which are not directly linked to the peak of the vertical movement dynamics, displayed bilateral differences of only about 3% when measured OG (Fig 2a). Thus, larger asymmetries occurred during movement phases incorporating high (almost maximal) loading of the affected structure, i.e. the knee, in the vertical direction. Lower knee flexion in leg-injured subjects has often been interpreted as a protective mechanism against knee pain [8, 34, 35]. It seems reasonable that, especially during vertical movements of the body, high forces acting on the knee may be associated with the occurrence of pain in the AL, causing avoidance behaviour that leads to bilateral asymmetries. Similarly, the heel-toe delay proved to be very sensitive to knee injury (asymmetry of 13%). Although there is not much force at the beginning of the heel touch, considerable force is built up during the transition to the toe touch (see the vertical acceleration in Fig 1). It is noteworthy that the force level at the time of toe touch is considerably lower in the AL compared to the NL (Fig 1) as the delay between heel touch and toe touch is shorter. At the same time, this shorter heel-toe delay in the AL presupposes a flatter foot-landing position. Previous research has assumed that flatter foot-landing positions might be perceived as more stable for runners [20]. Consequently, patients with knee problems may prefer a more stable landing position for their AL. Alternatively it may be speculated that although the ground reaction forces are not high at the time of the heel-touch, it is known that pronounced rear foot strike patterns increase external knee flexion moment [36]. Therefore, knee patients may prefer a fast transition to the toe touch in order to reduce knee flexion moment.

### Treadmill-induced changes in running symmetry

The reduction of the AL-NL asymmetry on the treadmill is highlighted by considering (a) the bilateral percentage differences (Fig 2b) and (b) the Symmetry Angle (Fig 3) for both ground conditions. As the results of these two analyses are very similar, we concentrate now on the SA. The KOA subjects of the current study displayed larger SAs OG for the knee flexion, vertical speed variance and heel-toe delay (SA>3) compared to the step and contact time (SA close to 1). Running on the treadmill induced significant reductions of SA between 28% and 80% for the contact time, knee flexion and the vertical speed variance (Fig 3). However, changes in the heel-toe delay and the step time were not significant (see Fig 3). For the heel-toe delay, large variations within and across subjects were observed making it difficult to find systematic adaptations (Fig 2). For the step time, the reason for the non-significance could be related to the fact that the step time is not under the permanent influence of high vertical forces. It therefore seems that symmetry was mainly altered on the TM during times of large ground reaction forces, which may seem counterintuitive at first glance. It is therefore important to analyse in detail how changes of the AL and the NL contributed to the enhanced symmetry. Interestingly, the enhanced symmetry on the TM mainly relied on adaptations of the NL. This was particularly visible for the contact time where the reductions on the TM were three and a half times larger for the NL than for the AL resulting in a drop of the SA of 80%. Changes in the NL also played the essential role for the vertical speed variance, where a trend towards reduction on the TM was only observed for the NL while the AL showed identical average values in both ground conditions (Fig 2). Thus, the adaptations that occur naturally on the TM such as flatter foot landing [20], reduced stance time, knee flexion and peak ground reaction force [14], help to restore symmetry as they go into the same direction for the healthy leg as the adaptations due to knee injury. In contrast, the AL seems to display a ceiling effect as the values hardly change when switching from OG to the TM.

The current results, showing large differences regarding the symmetry between OG and TM running, indicate that the type of ground greatly influences the sensitivity of detecting gait asymmetries. While all parameters except step time were significantly asymmetrical OG, none of these parameters was significantly different between AL and NL on the TM (see Fig 2). These observations corroborate the significant decrease of the SA on TM for the contact time, maximal knee flexion and vertical speed variance parameters. Thus, it seems that TM running masks KOA-induced gait asymmetries. This underlines the importance of taking into account ground conditions in order to make valid comparisons between studies with different ground types and when evaluating patients with KOA. Finally, with respect to rehabilitation and decreased asymmetry, the current study indicates that TM-training may be preferable over OG-training for KOA patients as the former restored gait symmetry.

The study had however some limitations: first of all, we only studied nine subjects which can reduce the statistical significance of the analysis. However, all subjects displayed the same behaviour between OG and TM for SA of four kinematic parameters. Secondly, only subjects with a light KOA injury could participate to the experiment, because they had to be able to run OG and on TM. A third limitation comes from the kinematic parameters that have been defined in the OG case. The ground reaction force signals were compared with the VICON foot markers behaviour to determine touch down and take-off times. If the reliability of the method could be verified for OG trials, this was not the case for the TM measurements. A treadmill allowing the measurement of the ground reaction force [37] would permit to fully validate the definition method of the kinematic parameters in the TM condition.

## 5. Conclusion

Our study showed that running gait symmetry in KOA patients was increased on a TM compared to OG. This was indicated by significant improvements in Symmetry Angle when switching from OG to the TM. Interestingly, the symmetry increase was mainly due to adaptations of the non-affected leg. The increased symmetry on TM for the investigated parameters stresses the importance of taking into account the ground type when analysing gait symmetry. In addition, the current results suggest that for the recovery of gait symmetry in KOA-patients, interventions on TM may be preferable.

## Supporting information

S1 Data (XLSX)
